# Teaching: the role of active manipulation of three-dimensional scatter plots in understanding the concept of confounding

**DOI:** 10.1186/1742-5573-2-6

**Published:** 2005-06-14

**Authors:** Cora MC Busstra, Rob Hartog, Pieter van 't Veer

**Affiliations:** 1 Division of Human Nutrition, Wageningen University, P. O. Box 8129, 6700 EV Wageningen, The Netherlands; 2 Wageningen Multi Media Research Centre, Wageningen University, Dreijenplein 2, 6703 HB Wageningen, The Netherlands

## Abstract

In teaching epidemiology, confounding is a difficult topic. The authors designed active learning objects (LO) based on manipulable three-dimensional (3D) plots to facilitate understanding of confounding. The 3D LOs help illustrate of how confounding can occur, how it generates bias and how to adjust for it. For the development of the LOs, guidelines were formulated based on epidemiology and theories of instructional design. These included integrating the conceptual and empirical aspects: the causal relationships believed to be operating in the study population (conceptual aspect) and data-oriented associations (empirical aspect). Other guidelines based on theories of instructional design included: actively engage the students, use visual methods when possible, and motivate the students about the importance of the topic. Students gave the method strong positive evaluations. Experts in epidemiology agreed that the 3D LOs apply generally accepted scientific views on confounding. Based on their experiences, the authors think that the 3D plots can be useful addition in the teaching of confounding. The article includes links and a downloadable file that provide a demonstration of the 3D LO-based teaching materials.

## Introduction

A major goal in teaching epidemiology is that students master the concept of confounding. They should understand when confounding may occur, how it can result in bias, and how to assess the presence of confounding and adjust for it.

As described by Rothman [1], "on the simplest level, confounding may be considered a confusion of effects. Specifically, the apparent effect of the exposure of interest is distorted because the effect of an extraneous factor is mistaken for or mixed with the actual exposure effect". (See Newman or Greenland for more fundamental definitions of confounding [2,3].) A confounding factor therefore must be: (1) a risk factor of the disease (in the unexposed), based on biological and epidemiological evidence, which requires information not included in the data; and (2) imbalanced between the exposure groups, which depends on the study design and population. In a dataset, these two criteria imply that a confounding factor must be associated with the disease and exposure. The third criterion for confounding is based on the causal relations between exposure, disease and confounding factor; this also requires information not included in the data. Rothman describes this third criterion as follows: (3) "A confounding factor must not be affected by the exposure or disease. In particular, it cannot be an intermediate step in the causal pathway between the exposure and the disease" [1].

Despite theoretical and practical work in our courses, problems in understanding confounding become clear when, in one of our courses, students analyze a dataset of a cross-sectional study. To do this, first the biological background of the exposure-outcome relation and potential confounding factors are presented. Next the students evaluate confounding using three plots: (a) of the crude association between exposure and outcome, (b) of the association between the potential confounding factor and the outcome and (c) of the association between the potential confounding factor and the exposure. Based on this information, the student must conclude whether confounding is present in the data and whether the crude association seen in the first plot provides a valid representation of the causal relationship between exposure and outcome in which the student is interested.

Communication with students indicated that knowledge of the criteria and their application to the dataset is not sufficient for understanding confounding. For example, it appeared difficult to imagine that confounding can invert the apparent direction of the effect of exposure. Several explanations of the unsatisfactory level of understanding can be put forward. One explanation is that students have to study the joint (three-dimensional) distribution of the exposure, outcome and confounding factor, but they have to use three separate (two-dimensional) plots instead of one three-dimensional plot. Obviously, simultaneously conceptualizing the three graphs requires complex cognitive processing and this could lead to cognitive overload. Another possible explanation is that most epidemiological textbooks tend to distinguish two aspects of confounding: In all textbooks, there is emphasis on *a priori *(prior to data collection) criteria for confounding (conceptual aspect) and on the evaluation of confounding by comparing crude and adjusted estimates (empirical aspect). The conceptual aspect focuses on background knowledge about the causal network that links exposure, outcome and potential confounders, which corresponds to the classical definition of confounding. The empirical aspect focuses on statistical associations within the data and corresponds to the collapsibility definition of confounding [2,3]. For students it seems difficult to understand how these two aspects are related.

To facilitate understanding of confounding, we developed digital learning objects (LOs) based on three-dimensional (3D) scatter plots. In the following, we describe the guidelines and requirements for the design of the 3D LOs, describe the 3D LOs and provide a hands-on example for the reader, and evaluate the results.

## Analysis

### Design process

Three-dimensional learning objects were designed for two courses: a BSc course (6 ECTS: European Credit Transfer System) which gives an introduction on study designs and the biases and an MSc course (6 ECTS), which focuses on data-analysis.

To direct the design process, guidelines were formulated, based on theories of instructional design (learning and teaching) and subject matter (content issues and learning goals). Students, teachers, and experts in epidemiology evaluated whether the requirements were fulfilled. In the next section, the guidelines and requirements that played a major role in the design of the 3D LOs are described. Emphasis is put on guidelines based on subject matter. Table 1 summarizes the guidelines, the requirements and the evaluators.

**Table 1:** Description of guidelines and requirements

Guidelines.	Requirements for the 3D LOs	Evaluation by
**Based on subject matter and learning goals**		
Use rotatable 3D plots.	- Students and experts perceive the 3D LOs as a valuable addition to the textbook.	Students and Experts
Integrate the conceptual and empirical aspect of confounding.	- Teachers confirm that the 3D LOs support the learning goals for confounding.	Experts
	- Experts in epidemiology confirm that the 3D LOs apply accepted scientific views on confounding.	Experts
	- Experts in epidemiology confirm that it is useful to use the 3D LOs in addition to epidemiological textbooks and lectures.	Experts
	- 80% of the students are able to answer exam questions (which integrate the conceptual and empirical approach) correctly.	Evaluation of exams
**Based on learning and instruction theories**		
Actively engage the students [26].	- Students feel that the elements in the 3D LOs that require them to become active learners help them to understand confounding.	Students
Visualize important concepts. [10,11].	- Students perceive the plots in the 3D LOs as a valuable addition to the textbook.	Students
	- Students feel that actively manipulating the 3D plots helps them to understand confounding.	Students
Motivate the students (based on ARCS model [27]): the LOs should:	- Students feel that the elements that require them to become active learners motivate them to study.	Students
- capture the Attention of the student,	- Students judge the material with at least a 4 (on a five-point scale).	Students
- be received as Relevant	- Students feel they learned from the 3D LOs.	Students
- induce Confidence and Satisfaction by students.	- The student is able to solve the exercises.	Students

### Design guidelines based on subject matter

#### Guideline: Use rotatable 3D plots

Proving an appropriate 3D illustration of the underlying 3D relationship, to help students to understand the concept of confounding, was the primary goal of this effort. Because epidemiological analyses usually deal with higher dimensional datasets, higher dimensional visualization techniques are used to design the 3D plots. These techniques aim at viewing several variables in the same representation, using computer-supported, interactive, visual representations of abstract data, to amplify cognition [4]. Several statistical software packages (such as SAS/insight and SPSS) offer three-dimensional visualization tools, like 3D scatter plots.

Some authors have recommended 3D scatter plots as tool for understanding statistical concepts [5] and as a tool for analyzing data [6,7]. Fox et al. stated that 3D scatter plots could be potentially useful when two-dimensional plots fail to reveal structure in the data, e.g. in case of certain kinds of clustering and non-linearity [8]. In addition, Yu found that subjects performed better in detecting outliers and examination of non-linear relationship using 3D plots than using 2D plots [9]. However, in these studies non-linear functions were used, so the conclusions should not be over-generalized to linear functions. In general, the use of a 3D plot instead of three 2D plots is helpful because a relationship between three variables may not be visible in 2D plots. A 3D plot, which can be rotated by the student, provides a better view of the distribution of the three variables in the 3D space. Furthermore, by projecting three-dimensional data on a two-dimensional plane it is possible to produce 2D plots to evaluate the criteria for confounding.

Furthermore, Larkin and Sweller suggest that, when images accompany text, understanding and retention of knowledge will generally improve [10,11]. Given our experience in teaching confounding, we expect that 3D data representation may also facilitate the understanding of confounding.

#### Guideline: Integrate the conceptual and empirical aspect of confounding

Some epidemiological textbooks distinguish the (a priori) conceptual and (data-based) empirical aspect explicitly [1,2,12-16] while others do so implicitly [17-23]. The conceptual aspect is usually illustrated by examples of exposures, diseases, confounding factors, and non-confounding covariates. Some textbooks summarize the criteria for confounding using causal path diagrams [12,14,20,21,23-25]. The empirical aspect is usually illustrated by examples of crude and adjusted data presented in tables [1,15,20,21] or graphs [22]. In this context, stratification and regression analysis are used as tools to assess the presence of confounding and to adjust for it. None of the examples we found in epidemiological textbooks illustrates how confounding can cause reversal of the apparent effect (i.e. the reversal of the sign of the association, the side of the null on which the effect lies) although some books do mention that it is a possibility.

Many students have trouble in connecting the two aspects of confounding when confronted with a real dataset. Therefore, we consider it important to integrate the two aspects of confounding in our teaching. This is achieved, in the 3D LOs, by visualizing that both aspects originate from the same 3D representation of the data. Our method integrates these aspects by illustrating that manipulating the association between the exposure and the confounder results in different crude associations (empirical aspect), although they are derived from the same underlying relationships (conceptual aspect).

### Design guidelines based on learning and instruction theories

The most important guidelines for the development of the 3D LOs, based on theories about learning and instruction, are summarized in this section.

#### Guidelines: Actively engage the student in studying confounding

The first guideline is to actively involve the student, because practice is believed to strengthen understanding [11,26]. In the 3D LOs, we will involve students in studying confounding with activities that include answering questions, performing simulations, and projecting data on one surface of the plot. In later applications of these methods we used self-tests to help clarify for students what was most important in the 3D LOs. Using these self-tests, the student could verify whether he understand the meaning of the different characteristics of the 3D LOs by interpreting some other examples of epidemiological data visualized in 3D plots.

#### Guidelines: Use visual methods when possible

A second guideline is to visualize important concepts. Besides visualizing the concept of confounding by using 3D plots, other visual methods are also used in the exercises that accompanied the 3D plots. For example, in the exercises, causal path diagrams are used to emphasize the causal relation between fiber intake, blood pressure and bodyweight.

#### Guidelines: Motivate the students

The last guideline is to motivate the students. Motivation is essential to learning. According to the ARCS model, four factors are essential to motivate the students: Instruction should capture the Attention of the student, it should be perceived as Relevant, and it should induce Confidence and Satisfaction [27]. From this principle, guidelines for the design of digital learning material were derived (see Table 1). The attention of the student is drawn by providing novelty (e.g., the 3D plots and several pictures). The relevance of the subject matter is shown by emphasizing the importance of the concept of confounding: the example used in the LOs illustrates the case where failure to adjust for confounding could lead to the conclusion that the effect of an exposure is in the opposite direction of the true relationship. Providing hints and gradually building up the difficulty of the exercises enhances students' confidence and satisfaction in understanding the concepts. For example, in the first 3D LO, several questions with hints are provided while in the third LO students are expected to explore the 3D plot by themselves. This third LO gives also the possibility to test skills that are attained in the first LOs.

### Requirements and evaluation

Students evaluated how well the teaching method fulfilled these guidelines in the BSc and MSc courses at our university, and in an international PhD course organized by our university. At our university students' perception of the quality of courses, course material and teachers was assessed with standard evaluation forms using agree-disagree questions on a five-point Likert scale. An average appreciation score of 3 on these evaluation forms is considered satisfactory while an average higher than 4 is considered excellent. The 3D LOs were specifically evaluated using such evaluation forms. In addition, exam results of students were analyzed to get an indication of their understanding of confounding.

For the evaluation with experts, evaluation forms with disagree-agree questions on a five-point Likert scale and free response questions were used. The experts worked through the 3D LOs and the exercises as if they were students. They were also asked to focus particularly on whether they think the 3D LOs apply accepted scientific views on confounding. Before this formal evaluation, three of our PhD students and two teachers evaluated the 3D LOs. This resulted in some minor improvements

### Description of the 3D LOs

The following is a description of one of the 3D LO-based lessons we used in our courses. It is based on data from (hypothetical) studies on the relation between fiber intake and blood pressure conducted in three different populations. Body weight is chosen as the potential confounding factor, because it is known to be a risk factor for high blood pressure. We constructed the example so that body weight is not an effect modifier. Each 3D LO starts with a rotatable 3D plot with the outcome (blood pressure) on the y-axis, exposure (fiber intake) on the x-axis, and the possible confounding factor (body weight) on the z-axis. In all the 3D LOs, the values of blood pressure, fiber intake and body weight are chosen so that body weight is a risk factor for high blood pressure and fiber intake is negatively associated with blood pressure. Only the association between fiber intake and body weight differs between the three plots.

**Figure 1:**
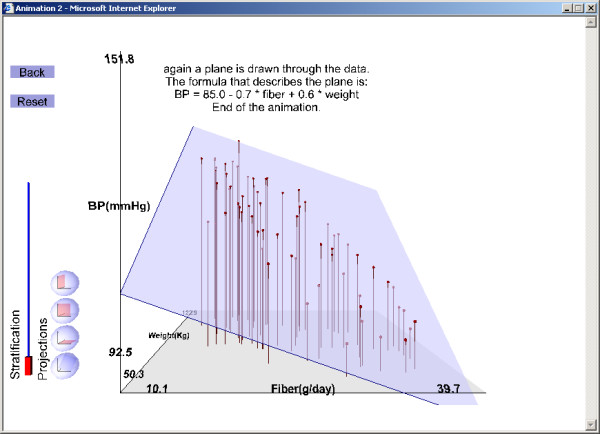
Illustrations of results from the example exercise (see text for instructions for running the exercise). Joint distribution of exposure (fiber intake), effect (high blood pressure), and potential confounder (body weight).

**Figure 2:**
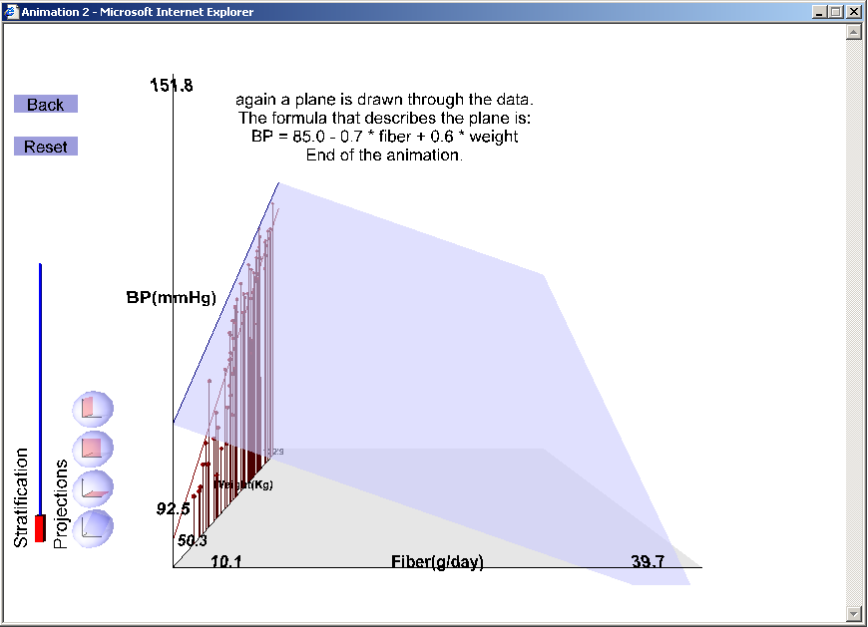
Illustration of results from the example exercise (see text for instructions on running the exercise). Projection of the data on the weight-blood pressure plane: weight risk is a risk factor for high blood pressure.

**Figure 3:**
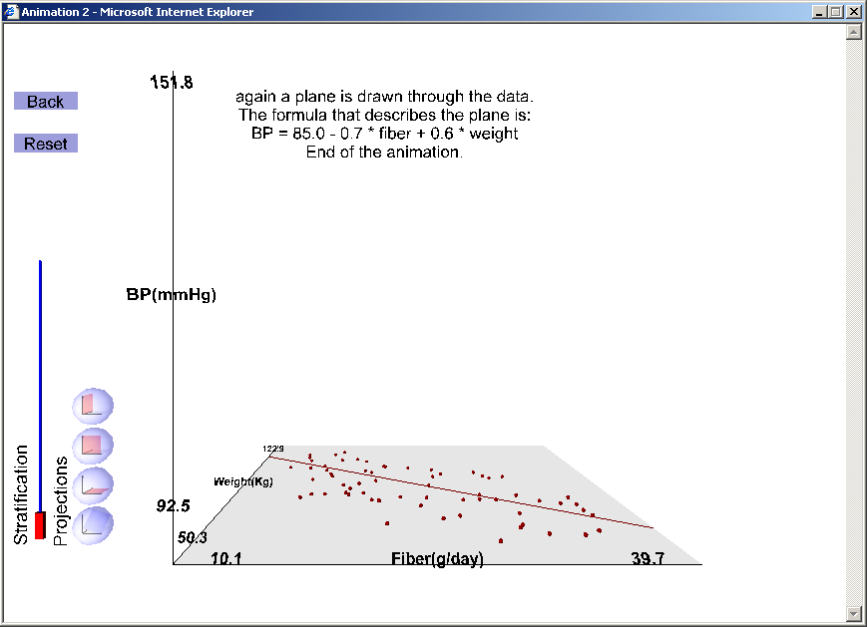
Illustration of results from the example exercise (see text for instructions on running the exercise). Projection of the data on the fiber intake–weight plane: fiber intake and weight are negatively associated.

**Figure 4:**
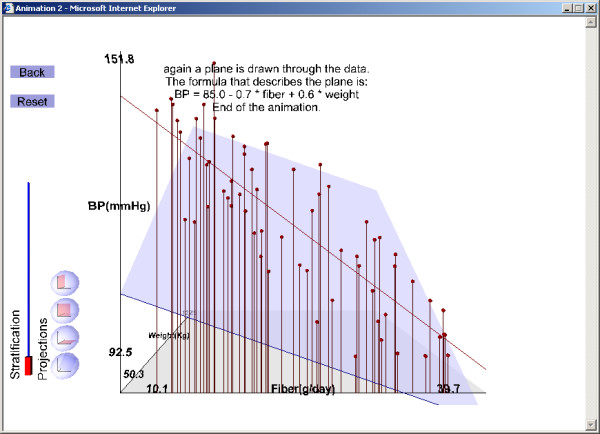
Illustrations of results from the example exercise (see text for instructions for running the exercise). Projection  of  the  data  on  the  fiber  intake–blood  pressure  plane:  the  crude  association  (the slope of the line) differs from the adjusted association (the slope of the plane).

In all plots the data can be projected on one side (plane) of the plot, so each plot illustrates:

1. The joint distribution of the three variables together: In all plots visualized by the linear plane fitted to the data (BP = β_0 _+ β_1 _* fiber intake + β_2 _* body weight + error) (Figure 1),

2. That body weight is a risk factor for high blood pressure (β_2_) (Figure 2),

3. The adjusted association between fiber intake and blood pressure (β_1_),

4. The association between fiber intake and body weight (differs between the LOs) (Figure 3),

5. The crude association between fiber intake and blood pressure, illustrated by a regression line through the projection of the data on the fiber-blood pressure side of the plot (Figure 4),

6. The association between fiber intake and blood pressure stratified for body weight (a slider can be used to highlight only data within a certain stratum of body weight).

The learning material consists of three parts, containing a 3D plot and some exercises. Figure 2 shows the main characteristics of the 3D plot as visualized in the second part of the learning material (the second LO).

**Figure 5:**
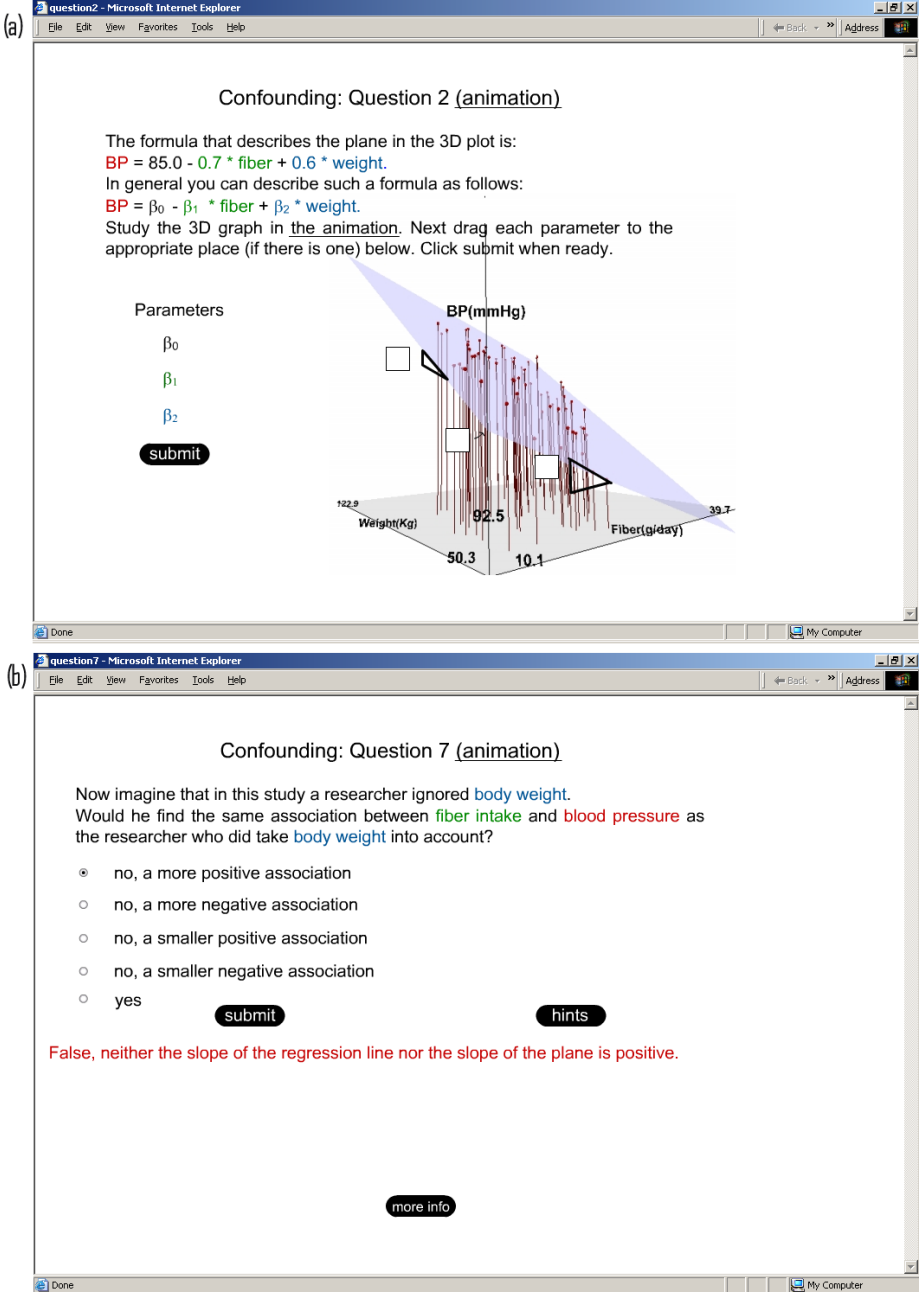
Examples of questions used to help students explore the characteristics of the 3D LOs

The 3D plot in the first LO represents data from a study in which fiber intake is independent of body weight. This LO illustrates the case where the apparent association between fiber and blood pressure is not confounded by the blood-pressure-increasing effect of body weight. In all LOs we assume that the effect of fiber intake on blood pressure is not mediated by body weight (criterion 3 for confounding [1]).

The second LO (Figure 1,2,3,4) and the third LO show that confounding arises when fiber intake and body weight are associated positively or negatively. For the second 3D LO, subjects with high fiber intake tend to have a lower body weight, perhaps because they are more health conscious. In the second 3D LO, the crude association (the slope of the line resulting from projecting the data to the fiber-blood pressure plane) differs from the adjusted association (the slope of the regression plane, β_1_) so body weight is a confounding factor (Figure 4). The reader can access the second 3D LO presented in this paper, as well as other examples, at our website [28]. (See endnote 1 for more information about the website and instructions on how to use the file published with this article which contains a version of what is on the website.)

In the third 3D LO, results of another (hypothetical) study shows how body weight reverses the apparent effect of fiber intake on blood pressure, when fiber intake and body weight are strongly positively associated.

### Practical experiences with the 3D LOs and results of evaluations

#### Evaluation by students

The 3D LOs are used in our BSc course (104 students, from which 100 filled out the evaluation forms), MSc course (in two subsequent years, in total 44 students) and an international PhD course organized by our university (19 students). Evaluation forms were used to assess the judgments of the students. As indicated in Table 2 the students judged the 3D LOs with a 3.7, 4.5 and 4.2 (on a five-point scale). The value of these student evaluations are limited by the lack of validation of the instrument, a clear definition of what the scores mean, and most importantly, the fact that few of these students had experience learning the material using other teaching tools, so they had nothing to compare this method to. Nevertheless, we interpret the scores as support for the value of this teaching method.

**Table 2:** Results of evaluation with students

	Mean score (% with a score of 4 or 5)		
	
Evaluation question*	BSc course (n = 100)	MSc course (n = 44)	International PhD course (n = 19)
1. The 3D plots help me to understand confounding.	3.6 (60)	4.4 (92)	4.2 (89)
2. It was useful to work with the 3D plots in addition to the lectures and textbook.	3.7 (68)	-^†^	-^†^
3. I enjoyed studying confounding using the 3D plots.	3.4 (53)	4.6 (100)	4.7 (100)
4. Active handling the 3D plots helps me to understand confounding.	3.5 (52)	4.5 (100)	4.2 (100)
5. The self-tests were useful.	- ^‡^	4.6 (100)	- ^‡^
6. Overall rating of the 3D plots (1 = poor to 5 = excellent).	3.7 (64)	4.5 (100)	4.2 (95)

*All questions were Disagree – Agree questions with a five-point Likert scale. As indicated an average score of 3 is considered satisfactory while an average higher than 4 is considered excellent.

^† ^In the MSc and PhD course this question was not included on the evaluation form because there was no additional learning material provided about confounding.

^‡ ^Self-tests were only available in the MSc and PhD course.

**Figure 6:**
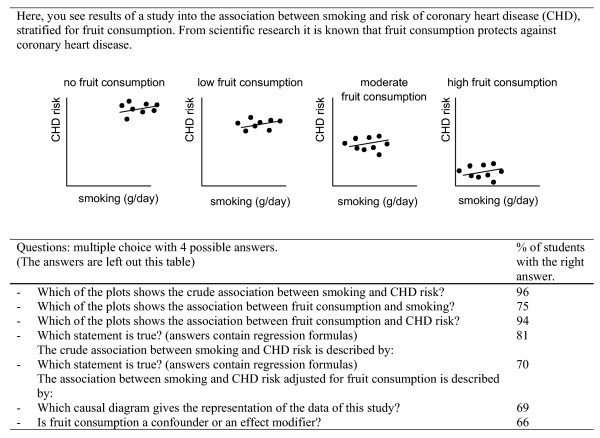
Example of exam question and summary of exam results

To get an indication of the level of competence attained by the students, exam results were analyzed. The exam questions were different for the BSc and MSc course. As indicated in Figure 6 the students scored well for the exam; for each question in the BSc course 66% or more of the students gave the right answer. The questions about the integration of the conceptual and empirical aspect of confounding appear the most difficult ones (question 6 and 7). In the MSc course, in two multiple-choice questions descriptions of epidemiological studies must be combined with plots that show the data of the studies. On these questions, respectively 83 and 75% of the students gave the correct answer. Although the same exam questions were not asked in the past, this rating is considerably better than the results from similar exam questions on the same topic that were asked in the past.

Illustration of the usefulness of the method to the students came in the MSc course, where students further practiced with 3D plots during the analysis of a cross-sectional study. Most of the students took advantage of the opportunity to consult the 3D LOs again during the data-analysis. From our experiences in previous years, it seems that during this MSc course students who were taught using the 3D LOs had a better understanding the concept of confounding and multiple regression as a method to adjust for confounding than previous years (though we concede that this evaluation suffers from the usual problems of non-blinded evaluators who are invested in the outcome). Students asked questions that are more advanced. For instance, many students extrapolated the method to effect modification by describing how a 3D plot would look like in the presence of effect modification.

Since the courses in which the 3D LOs were used and similar courses in which they were not used differ from year to year with respect to specific topics, learning material, form of the exam, number of students, prior knowledge of students, etc., it is not possible to investigate precisely the effect of the 3D LOs (as it would had we been able to do a clean and large scale randomized study). This is a well-known challenge in educational research [29]. Therefore, rather than relying too much on the students' demonstrated learning and own evaluations of the methods, we base much of our evaluation on the more indirect method of assessing how well 3D LOs fulfilled the above guidelines and how experts evaluated them.

#### Evaluation by experts in epidemiology

Eight experts in epidemiology reviewed the 3D LOs; seven were teachers at Dutch universities and one at a non-Dutch university. Six of them filled in the evaluation form while two only responded by giving a general opinion about the 3D LOs. The experts were not involved in the design of or teaching using the 3D LOs. Table 4 summarizes the scores on the evaluation questions. In addition, the experts responded to some open-ended questions. The results suggest that the experts agree that the 3D LOs apply generally accepted scientific views on confounding and should enhance understanding of confounding. However, two experts expressed concern that the 3D LOs would not be helpful for some students who have difficulties with interpreting 3D objects. Three experts suggested that we develop additional learning material explaining the difference between confounding and effect modification. There were also suggestions that the issue of causality in relation to the third criterion [1] for confounding needed further explanation, which we have added (though this change came subsequent to the students' experience with the learning material).

**Table 4:** Evaluation of the 3D LOs by experts in epidemiology

Evaluation question*	Mean Score (n = 6)
1. I think the students like the module.	4.3
2. The questions in this modules where clear and understandable	4.8
3. It is useful that the 3D plots are rotatable	3.0
4. The questions in this module are useful	4.8
5. I think that this module applies general accepted scientific views on confounding	4.5
6. I think that the use of 3D plots enhanced understanding of confounding by students	4.0
7. I think that this modules provides a useful addition to epidemiological textbooks and lectures	4.2
8. I think that this module stimulated the student to study confounding	3.8
9. I think that this module is useful in my own course.	3.8
10. Overall rating of the module.	3.8

*All questions were Disagree – Agree questions with a five-point Likert scale.

## Conclusion

Recently, other graphical approaches to teaching confounding have been described [30,31]. Unlike our 3D LOs, these approaches address confounding without the use of multivariate regression techniques. Therefore, the approaches could be useful to introduce the concept of confounding and to make the students aware of the importance of considering possible confounders. These approaches do not directly address the relation between the criteria for confounding (conceptual aspect) and the effect of the confounder on the studied exposure-outcome relation (empirical aspect), as do the 3D LOs. Thus, the 3D LOs seem to be more useful at an intermediate level, preparing the students for epidemiological data analysis. Therefore, we think the approaches could complement each other.

Teaching tools using 3D plots are potentially useful in illustrating effect modification, non-linearity in datasets [8], and other relationships of three variables. We plan to design additional learning material contrasting confounding and effect modification. In addition, 3D plots can be useful in teaching other epidemiological principles. For example, how measurement errors in the confounding factor, exposure variable, or outcome variable can lead to, respectively, residual confounding, bias toward the null, or decrease of precision. We will make revisions of the current method and additions of other concepts in our 3D LOs available at our website [28].

Our first experience with the 3D LOs indicate that the integration of the conceptual and the empirical aspect of confounding stimulate the student to think beyond confounding. Although it might be possible that the 3D LOs will not be helpful for some students (e.g. students who have difficulties with interpreting 3D objects) we think that, based on our experiences, the 3D LOs can provide a valuable addition to standard epidemiological textbooks and other graphical presentations of confounding for most students.

## Endnotes

1. To ensure the existence of a permanent archive, the website that contains the example emphasized in this article has been published with the article as an additional file (however, the website is easier to use, more extensive, and will contain subsequent versions of the software, and thus we recommend readers access it at  if possible rather than using the additional file). To use the additional file, download the .zip file, unzip it to a folder, and run (double click on) index.html.

Note that to run either the web or local version of this demo requires the Macromedia Flash player browser plug-in, which you probably have, as well as a plug-in for viewing 3D images (Cortona from Parallel Graphics) that you will likely need to install. These are free and the index page contains links that will let you install them. We apologize that in its present form, our software will not work with all browsers, security configurations, etc. We recommend the use of Microsoft Internet Explorer and it will be necessary to turn off pop-up blockers. The index page contains a link to check your system's compatibility.

## List of abbreviations

2D, Two-dimensional.

3D, Three-dimensional.

BSc, Bachelor of Science.

ECTS, European Credit Transfer System.

LO, learning object.

MSc, Master of Science.

PhD, Postdoctoral.

## Competing interests

The author(s) declare that they have no competing interests.

## Authors' contributions

MCB designed, developed and evaluated the 3D LOs and led the writing of the manuscript, but all three authors contributed to editing and revision. RH initiated the project and provided the initial arguments for investing in the development of some form of 3D visualization; he reviewed the LOs from an educational point of view. PvtV contributed in the design of the 3D LOs and reviewed the epidemiological content of the 3D LOs.
